# Gastric Pseudomelanosis: An Uncommon Finding

**DOI:** 10.1155/2022/6290510

**Published:** 2022-07-30

**Authors:** John Samies, Ruchit N. Shah, Michelle Pramick, Alberto Unzueta

**Affiliations:** ^1^Division of Hospital Medicine, Geisinger Medical Center, Danville, PA, USA; ^2^Division of Gastroenterology and Hepatology, Geisinger Medical Center, Danville, PA, USA; ^3^Division of Pathology, Geisinger Medical Center, Danville, PA, USA

## Abstract

Gastric pseudomelanosis is a benign condition that has been characterized by pigment deposition within subepithelial macrophages inside the stomach wall. Occurrence of the condition is rare, with pseudomelanosis occurring more often within the duodenal mucosa. Few cases have reported pseudomelanosis within the gastric mucosa. We report a case of an 86-year-old female who presented with worsening iron deficiency anemia concerning for gastrointestinal bleeding. Her endoscopic evaluation was remarkable for a speckled pattern of dark pigmentation within the stomach, confirmed to be pseudomelanosis by histologic evaluation with pigment showing positive staining for iron.

## 1. Introduction

Pseudomelanosis of the upper gastrointestinal tract is a rare discovery, with the condition typically being noted as an incidental finding on endoscopic evaluation. The condition presents as speckled dark mucosal pigmentation. We report a case of an 86-year-old female who required evaluation for worsening anemia concerns and suspected gastrointestinal bleeding. Her evaluation was remarkable for pseudomelanosis of the stomach, confirmed with biopsy.

## 2. Case

An 86-year-old female with a medical history significant for gastroesophageal reflux, stage IV chronic kidney disease, chronic obstructive pulmonary disease, prior cerebrovascular accident, hypertension treated with verapamil, hyperlipidemia, and iron deficiency anemia was admitted to the hospital for further evaluation of daily vomiting episodes over a 2-week period. The patient was found to have worsening anemia concerns, with an admission hemoglobin of 7.2 g/dL (RR: 14.0–16.8 g/dL).

The patient was noted to have had recent workup 3 weeks prior for symptomatic anemia at an outside institution, with upper endoscopy only significant for mild gastritis and colonoscopy with normal anatomy.

**Figure 1:**
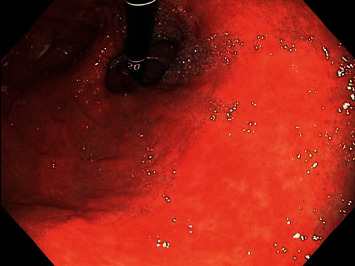
Gastric fundus demonstrating discolored and friable mucosa with few scattered areas of speckled dark mucosal pigmentation (endoscopy image).

**Figure 2:**
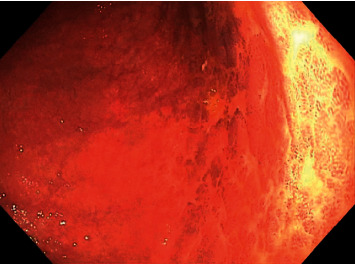
Gastric body demonstrating speckled dark mucosal pigmentation (endoscopy image).

**Figure 3:**
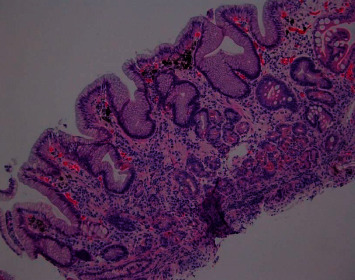
Gastric mucosa with inactive chronic gastritis, foveolar hyperplasia, mucosal iron deposition, intestinal metaplasia, and superficial pigment deposition, suggestive of pseudomelanosis. H&E stain, 100x.

**Figure 4:**
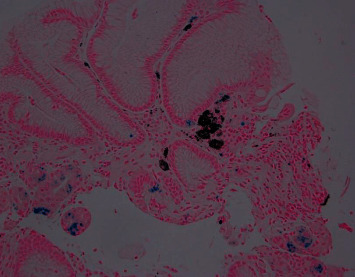
Gastric mucosa. An iron special stain highlights mucosal iron deposition. Subepithelial aggregates of dark pigment are also seen. Iron stain, 200x.

On admission, the patient denied abdominal pain or hematemesis, but did report occasional dark colored stools in the setting of oral iron supplementation and daily aspirin use. Given suspected gastrointestinal bleed, the patient was provided with a blood transfusion of 1 unit of packed red blood cells and subsequently underwent upper endoscopy with video capsule placement. Patient's updated endoscopic evaluation revealed discolored and friable mucosa in the gastric fundus and gastric body (Figures 1, 2). Video capsule study revealed pigmented duodenal mucosa and no active bleed. Biopsies of the stomach were obtained, which revealed chronic gastritis, mucosal iron deposition, and superficial pigment deposition suggestive of pseudomelanosis (Figures 3, 4). Biopsy immunostaining for *H. pylori* was negative. The patient did well after endoscopic evaluation and was subsequently deemed stable for discharge from the hospital after a short period of close monitoring. She was continued on iron supplementation at time of discharge due to her significant anemia concerns. No follow-up endoscopy was deemed necessary with her condition, as her hemoglobin remained stable after discharge and patient did not wish for any additional testing.

## 3. Discussion

Gastric pseudomelanosis is a rare condition that presents as speckled black pigmentation in the gastric mucosa [1, 2]. The dark pigment accumulation visualized on endoscopy corresponds to subepithelial pigment deposition within macrophages of the lamina propria histologically. The pigmentation results from several different compounds depending on the etiology. Deposition of lipomelanin, ceroid, iron sulfide, and hemosiderin has been implicated in the formation of this endoscopic appearance [1, 2].

Gastric pseudomelanosis is found more often in patients noted to have a history of hypertension, chronic renal insufficiency, and oral iron supplementation [3]. Conditions also noted to increase incidence of gastric pseudomelanosis include diabetes mellitus and history of upper gastrointestinal bleeding. Certain medications have also been implicated, including hydralazine, thiazide diuretics, furosemide, and beta-blockers [1–5]. Although pseudomelanosis has been reported in children, most cases are noted to occur in older adult females [3–5]. The exact mechanism that may precipitate gastric pseudomelanosis has not been elucidated. Specific treatment is not indicated for drug-induced etiologies. However, it is important to address any underlying causes of iron overload.

The diagnosis of pseudomelanosis of the upper gastrointestinal tract may become more common over time due to the aging patient population, especially as more patients develop the associated medical comorbidities that have been implicated in the formation of this condition. Physicians should be aware of pseudomelanosis to expedite diagnosis and to provide cost-effective care by minimizing further testing.
